# Pain Severity and Vitamin D Deficiency in IBD Patients

**DOI:** 10.3390/nu12010026

**Published:** 2019-12-20

**Authors:** Svein Oskar Frigstad, Marte Lie Høivik, Jørgen Jahnsen, Milada Cvancarova, Tore Grimstad, Ingrid Prytz Berset, Gert Huppertz-Hauss, Øistein Hovde, Tomm Bernklev, Bjørn Moum, Lars-Petter Jelsness-Jørgensen

**Affiliations:** 1 Department of Research, Østfold Hospital, 1714 Grålum, Norway; 2 Department of Medicine, Vestre Viken Bærum Hospital, 1346 Gjettum, Norway; 3 Institute of Clinical Medicine, University of Oslo, 0318 Oslo, Norway; marte.lie.hoivik@gmail.com (M.L.H.); jorgen.jahnsen@medisin.uio.no (J.J.); Ingrid.Prytz.Berset@helse-mr.no (I.P.B.); oistein.hovde@sykehuset-innlandet.no (Ø.H.); tbernk@siv.no (T.B.); bjorn.moum@medisin.uio.no (B.M.); 4 Department of Gastroenterology, Oslo University Hospital, 0424 Oslo, Norway; miladacv@medisin.uio.no; 5 Department of Gastroenterology, Akershus University Hospital, 1478 Nordbyhagen, Norway; 6 Department of Biostatistics, Oslo Metropolitan University, 0130 Oslo, Norway; 7 Department of Gastroenterology, Stavanger University Hospital, 4068 Stavanger, Norway; tore.bjorn.grimstad@sus.no; 8 Department of Medicine, Telemark Hospital, 3710 Skien, Norway; hhge@sthf.no; 9 Department of Medicine, Innlandet Hospital, 2819 Gjøvik, Norway; 10 Department of Research and Development, Vestfold Hospital, 3103 Tønsberg, Norway; 11 Department of Gastroenterology, Østfold Hospital, 1714 Grålum, Norway; lars.p.jelsness-jorgensen@hiof.no; 12 Department of Health Sciences, Østfold University College, 1757 Halden, Norway

**Keywords:** vitamin D deficiency, pain severity, patient reported outcomes

## Abstract

*Background*: Pain and vitamin D deficiency are common in inflammatory bowel disease (IBD). Disease activity, fatigue, frequent relapses, prior surgery and psychological factors all seem to influence the experience of pain in IBD. Vitamin D deficiency has been associated with muscle and skeletal pain. This study aimed to determine whether there is an association between vitamin D deficiency and severity of pain in patients with IBD, and to investigate the influence of other socio-demographic and psychological variables on the experience of pain. *Methods*: Patients with IBD were recruited from nine hospitals in Norway in a multicenter cross-sectional study. The Brief Pain Inventory (BPI) questionnaire was used to measure pain. Disease activity was assessed using clinical disease activity indices, C-reactive protein (CRP) and fecal calprotectin. Regression models were fitted to explore a possible association between 25-hydroxyvitamin D and pain severity. *Results*: Of 407 patients included in the analyses, 229 (56%) had Crohn’s disease (CD) and 178 (44%) had ulcerative colitis (UC). Vitamin D deficiency was present in half (203/407) of patients. Presence of pain was reported by 76% (309/407). More severe pain was associated with female gender and increased disease activity scores, but not with increased CRP or fecal calprotectin. In CD, patients without prior intra-abdominal surgery reported more severe pain. In multivariate analyses, there was no association between 25-hydroxyvitamin D and pain severity. *Conclusions*: In this study, no significant association between pain severity and vitamin D deficiency was revealed in patients with IBD.

## 1. Introduction

Inflammatory bowel diseases (IBD), including ulcerative colitis (UC) and Crohn’s disease (CD), are long-term conditions that involve the inflammation of the gastrointestinal tract [1,2,3]. Symptoms of IBD include diarrhea with or without blood, fatigue and abdominal pain [2]. Abdominal pain is frequently reported in IBD, and may be caused by, e.g., gut distension, obstruction and inflammation [4]. Many patients are also troubled by extraintestinal manifestations that may be associated with pain, such as arthralgia, skin lesions, oral aphthous lesions and bone disease [5,6].

A relapsing course of IBD is seen in many patients, which may cause the intermittent exacerbation of abdominal pain [2]. Furthermore, sensory pathways are sensitized during active inflammation, which may lead to persistent changes in the experience of pain [4]. Despite efficacious anti-inflammatory treatment and clinical remission, more than one-third of IBD patients continue to experience pain [4].

Modulating factors, including different psychological factors, may significantly contribute to the clinical manifestation and severity of pain [7,8,9]. Emotional problems and inadequate coping mechanisms have been associated with development of chronic pain syndromes [4]. Furthermore, the use of analgesics, including opioids, has been reported to be considerable in some patients with IBD, and an association with poor prognosis has been suggested—mainly due to use of analgesics being a surrogate marker of inflammatory activity and penetrating disease [10].

In addition to abdominal pain, many IBD patients report lower back pain and other sites of chronic pain that do not always correspond to disease activity [11]. Abdominal pain, joint pain and lower back pain have all been associated with reduced health-related quality of life in IBD [12,13]. Fatigue—a highly prevalent symptom in IBD—appears to be more frequent in patients with pain, and pain should therefore be considered when reporting fatigue in IBD patients [13,14].

Vitamin D receptors are expressed in many tissues including skin, bone, muscles, endocrine tissues and the immune system, and thus many organs require vitamin D for optimal function and, accordingly, vitamin D may influence mechanisms of pain [15]. Several studies have shown that vitamin D deficiency is common in IBD patients, and even more frequent than in the general population [16,17,18,19,20,21]. In general practice, vitamin D deficiency has been associated with musculoskeletal pain and headaches [22]. In IBD, however, no relationship between pain and vitamin D has been established.

The primary aim of this study was to determine whether there is an association between vitamin D deficiency and severity of pain in patients with IBD. The secondary aims were to investigate the influence of selected socio-demographic and psychological variables on the experience of pain.

## 2. Materials and Methods

### 2.1. Ethical Considerations

The study was approved by the Regional Committee for Medical and Health Research Ethics (2012/845/REK). All the study participants gave written, informed consent before their inclusion in the study, and the study was performed in accordance with the Declaration of Helsinki.

### 2.2. Study Setting and Population

Patients were recruited between March 2013 and April 2014 from nine hospitals in south-eastern and western Norway for this observational, multicenter cross-sectional study. The inclusion criteria were: age ≥ 18 years, a verified diagnosis of IBD based on endoscopic, biochemical and histological findings according to the Lennard-Jones criteria [23], and the ability to read and understand Norwegian and to give written, informed consent.

### 2.3. Clinical, Socio-Demographic and Laboratory Variables

Data were collected using patient interviews, medical records, and laboratory tests. All data were collected at inclusion.

Serum 25-hydroxyvitamin D [25(OH) D] was analyzed by one central accredited laboratory using liquid chromatography tandem mass spectrometry (LC-MS/MS). The method used has been compared to the LC-MS/MS method used in the vitamin D standardizing program, where it showed good compliance [24]. Vitamin D deficiency was defined as 25(OH) D concentration <50 nmol/L, and vitamin D insufficiency was defined as a 25(OH) D concentration of 50–75 nmol/L [15]. Further details on the method can be found in Appendix A.

All other biochemical analyses were performed at the local laboratories at the participating hospitals. C-reactive protein (CRP) level of ≥5 mg/L was set as a cut-off for active inflammation [25,26].

The stool samples for the measurement of fecal calprotectin were sent by post and analyzed with Calpro Easy Extract (Calpro AS, Lysaker, Norway). A fecal calprotectin level of ≥100 mg/kg was used to indicate mucosal inflammation [27].

Clinical disease activity was measured with the Simple Clinical Colitis Activity Index (SCCAI) for UC, and the Harvey Bradshaw Index (HBI) for CD [28,29]. For UC, an SCCAI score ≥5 indicated active disease [25,26,30,31], and in CD, an HBI score ≥5 was used to define active disease [28,32].

### 2.4. Assessment of Pain and Psychological Factors

Pain was measured using the Brief Pain Inventory (BPI) [33]. The BPI is suitable for clinical and epidemiological purposes and has been translated into Norwegian and validated, including validation in IBD patients [34,35]. The BPI was designed to measure both the intensity of pain and the interference of pain on daily activities. The pain intensity assessment consists of four items that are scored on a scale from 0 (no pain) to 10 (worst possible pain), and a pain severity score is calculated from the mean of the four items. A mean severity score of 1–3 was classified as mild pain, 4–6 as moderate pain and >7 as severe pain [35]. The assessment of pain interference during the previous week consisted of seven criteria that were scored from 0 (no interference) to 10 (complete interference), and a pain impairment score was calculated from the mean of the seven criteria [33].

Depressive and anxiety symptoms were measured with the Hospital Anxiety and Depression Scale (HADS), which has been translated into Norwegian and validated [36]. HADS is a 14-item questionnaire, designed to reveal potential depressive (7 items) and anxiety (7 items) symptoms. Each item is scored from 0 to 3, and thus the total score ranges from 0 to 21 for depression or anxiety, respectively [36]. A higher score indicates an increased level of reported symptoms. In this study, depressive mood was defined as a HADS-Depression (HADS-D) subscore ≥8 and presence of clinically relevant anxiety was defined as a HADS-Anxiety (HADS-A) subscore ≥8. These scores are the most relevant for screening of psychological symptoms in chronic disease [37].

### 2.5. Statistical Analyses

Normally distributed continuous variables are presented with means and standard deviation, while variables with skewed distributions are described with medians and ranges. Crude differences between pairs of continuous variables were analyzed using Student’s *t*-test when normally distributed, otherwise a non-parametric test was used (Kruskal–Wallis test). The associations between pairs of categorical variables were analyzed with a Chi-square test, and for associations between continuous variables, the Pearson correlation test was used. To explore possible associations between the selected variables and severity of pain, ordinal logistic regression models were fitted, using pain severity as the dependent variable. The variables with a *p*-value < 0.1 in the univariate analyses were entered into the final multivariate models. Those variables with *p*-values < 0.05 were considered statistically significant in the multivariate analyses. All tests were two-sided. The study was considered exploratory, so no correction for multiple testing was done. IBM SPSS Statistics for Windows version 25.0 (IBM Corp. Armonk, NY, USA) was used for all statistical analyses.

### 2.6. Study Outcomes

The main outcome variables were severity of pain and vitamin D deficiency. For the statistical analyses, three patient groups were formed: (i) no pain, (ii) mild pain and (iii) moderate or severe pain. These groupings were considered the most relevant approach from a clinical perspective, but also statistically, as relatively few patients reported moderate and severe pain. Additional outcomes included selected socio-demographic and psychological variables. The guidelines of the STROBE statement have been adopted.

## 3. Results

In total, 452 patients were invited to participate; of these, 414 (92%) gave written consent. Seven patients were excluded due to missing data from questionnaires. Thus, 407 patients were available for the analyses, of which 229 (56%) had CD and 178 (44%) had UC. There were no statistically significant differences between the UC and CD patients in terms of age or gender. The CD patients had significantly longer disease duration than UC patients (median 11 vs. 6 years, *p* < 0.01). Furthermore, more CD patients were receiving treatment with biologics (50% vs. 29%, *p* < 0.01). Approximately half (203/407) of the patients had vitamin D deficiency, and around one-third (135/407) were using low-dose vitamin D supplementation. Furthermore, vitamin D deficiency was shown to be associated with increased clinical disease activity, a relapsing disease course, and higher inflammatory activity [20]. Further details are provided in Table 1 and Table 2.

Presence of pain was reported by 76% (309/407) of the patients, most commonly in the abdomen, back and joints (Table 3). The mean scores for severity of pain were similar in patients with UC and CD (mean 8.2 ± 7.5 vs. 7.5 ± 6.4, *p* = 0.35). Current smokers reported more pain, but this was not statistically significant, and the number of daily smokers in this sample was low. In univariate analyses, increased symptoms of depression and anxiety were associated with pain severity in UC patients, but not in patients with CD.

In the multivariate analysis, adjusted for gender, vitamin D level, and symptoms of depression and anxiety, pain severity was associated with elevated disease activity scores in UC and in CD. However, pain severity was not associated with objective inflammatory markers CRP or fecal calprotectin. CD patients with no prior intra-abdominal surgery reported pain that was significantly more severe than pain reported by those who had undergone surgery. Pain severity was significantly associated with the female gender in UC and in CD. The analyses are summarized in Table 4 and Table 5.

The mean scores for pain interference were similar in patients with UC and those with CD (mean 14.0 ± 15.2 vs. 13.9 ± 15.4, *p* = 0.96). More severe pain was associated with increased pain interference in daily activities in both UC (Kruskal–Wallis, *p* < 0.01) and CD (Kruskal–Wallis, *p* < 0.01). There was no significant association between vitamin D deficiency and pain interference in daily activities in either UC (Pearson, r = −0.11, *p* = 0.16) or CD (Pearson, r = −0.05, *p* = 0.42).

## 4. Discussion

In this cross-sectional study of IBD patients, neither pain severity nor pain interference with daily activities was significantly associated with vitamin D deficiency. However, severity of pain was associated with elevated disease activity scores in UC and in CD, but not with objective inflammatory markers such as CRP and fecal calprotectin. CD patients without prior intra-abdominal surgery reported more severe pain. Pain severity was significantly associated with the female gender, but surprisingly not with a higher burden of depressive or anxiety symptoms.

To our knowledge, no previous studies have investigated whether vitamin D deficiency is associated with pain severity in IBD patients. Vitamin D deficiency is, however, known to cause musculoskeletal pain in other conditions, e.g., osteomalacia [15]. Furthermore, a Norwegian study from a multi-ethnic general practice population, found that vitamin D deficiency was associated with more severe pain in patients with a primary complaint of either headache or musculoskeletal pain [22]. These findings may suggest a role of vitamin D deficiency in the experience of pain. Insufficient vitamin D in IBD may result in increased severity of pain in some patients, due to a more active disease with a higher burden of symptoms, but a heightened sensitivity to visceral pain cannot be ruled out [38,39]. One possible mechanism for such an effect may be that vitamin D inhibits the mediators of inflammatory pain—e.g., Prostaglandin E—as has been shown in other inflammatory diseases [40,41].

In the current study, no association between the severity of pain and vitamin D deficiency was found. Many patients were treated with biologics, especially in CD, and this may represent a selection bias towards patients with more severe disease, who may be expected to have a higher burden of abdominal pain. Conversely, effective medical and surgical treatment may have improved pain scores in many patients. Effective treatment may, therefore, have diminished the potential effect of vitamin D deficiency on the experience and reporting of pain in patients with more active disease. In clinical practice, however, it is not always the case that intra-abdominal surgery reduces the severity of abdominal pain over time, as many patients develop abdominal adhesions, especially if multiple surgeries are performed.

In interventional studies assessing the effects of vitamin D supplementation on pain severity, the results are conflicting [42,43]. In a Norwegian general practice study, vitamin D supplementation in patients with vitamin D deficiencies showed no significant difference in the severity of musculoskeletal pain or headache compared to placebo, but numerically lower pain scores were reported in both groups on follow-up [44]. Vitamin D supplementation has, however, been shown to be associated with the improvement of severity of pain in chronic musculoskeletal pain among immigrants with vitamin D deficiencies, and in children with growing pains—regardless of their vitamin D status [45,46]. It was beyond the scope of this study to assess such effects, but as previously mentioned, it has been suggested that insufficient vitamin D predisposes patients to deep muscle hypersensitivity [39].

Vitamin D also has effects on immune responses, which may reduce intestinal inflammation [15,47,48]. Pain is more often reported in patients with active disease, and it could be speculated that a vitamin D deficiency may contribute to disease activity, and consequently increase the experience of pain. In the current study, our data revealed an association between pain severity and higher disease activity scores, regardless of diagnosis, but not with objective inflammatory markers (CRP and fecal calprotectin). Although inflammation is important, this suggests that other factors may play a role, and that the total burden of symptoms may contribute relatively more to the experience of pain than the intestinal inflammation alone.

In this regard, the high prevalence of symptoms of irritable bowel disease in patients with IBD is certainly a factor to consider, since pain is a hallmark among these symptoms [4,49,50]. In UC, such symptoms have been shown to be as common in remission as in active inflammation [51]. Furthermore, an American study on abdominal pain in patients with UC also concluded that many patients report abdominal pain in the absence of active inflammation [52]. Due to symptoms not always representing active inflammation, clinical indices may not always correspond to disease activity.

Various symptoms not directly related to intestinal inflammation have been found to impact on patient-reported outcomes and may, therefore, be of importance in patient management. A recent review has demonstrated several psychological factors to be associated with pain in IBD patients and that such factors remain important regardless of disease activity [9]. Although we found that depressive and anxiety symptoms were not independently associated with pain severity in multivariate analyses, these symptoms may be of importance in some patients [7,9].

We have previously shown that pain severity was not significantly associated with fatigue [14]. Pain may, however, be of importance for some patients troubled by fatigue. A Swedish study has shown that patients with more severe gastrointestinal symptoms, including pain, report more fatigue [53]. Furthermore, patients with severe fatigue are more likely to suffer from mood disorders such as depression, and there is an association between fatigue and psychological comorbidity that may be important in the understanding of chronic pain in patients with IBD [54,55].

In the present study, higher pain severity scores were significantly associated with the female gender. This is in accordance with other studies showing that female patients with IBD may be at particular risk of experiencing and reporting pain [52,56]. Smoking may be of less relevance as a contributing factor in the Norwegian IBD population, where there are relatively few daily smokers. The use of analgesics is known to be associated with more severe disease, but further assessment of the role of analgesics on pain severity was not carried out in this study. Since few patients were receiving corticosteroid treatment, analyses of the effect of steroids on the experience of pain could not be performed.

A limitation of our study is the cross-sectional design, which meant that no causal inference was possible. The major strengths are the relatively large sample size, the high level of completeness and the lack of selection bias. The patients were recruited from several hospitals and all approached patients were equally likely to be included, thus our sample is considered representative of IBD patients treated in specialist care settings.

The duration of disease was longer in CD patients as compared to UC patients, and this may have influenced patient-reported outcomes such as depressive symptoms, anxiety and pain. It is not known how disease duration influences these outcomes in chronic disease, but it may lead to under-reporting, as patients may become accustomed to living with symptoms over time (i.e., response shift). With self-reporting of symptoms, there is also a risk of recall bias.

## 5. Conclusions

Pain is commonly reported in IBD patients and is associated with clinical disease activity and the female gender. Our study did not reveal any significant association between vitamin D deficiency and pain severity, supporting a bio-psycho-social approach to the understanding of pain in IBD patients. Further studies are needed to explore whether better pain management may improve outcomes, and to further elaborate on the possible importance of coping mechanisms and psychological factors beyond depression and anxiety.

## Figures and Tables

**Table 1:** Socio-demographic and Clinical Data.

	UC (*n* = 178)	CD (*n* = 229)
Age median, years (range)	40 (18–76)	40 (18–77)
Female gender, *n* (%)	87 (48)	114 (50)
Disease duration median, years (range)	6 (0–46)	11 (0–50)
Smoking, *n* (%)	16 (9)	34 (15)
UC extent, Montreal classification, *n* (%)		
E1–Proctitis	20 (11)	
E2–Left-sided colitis	58 (32)	
E3–Extensive colitis	102 (57)	
CD localization, Montreal classification, *n* (%)		
L1–Terminal ileum		75 (32)
L2–Colon		47 (20)
L3–Ileocolon		76 (33)
L4–Upper gastrointestinal		32 (14)
Disease activity, *n* (%)		
SCCAI ≥5	53 (29)	
HBI ≥5		106 (46)
Fecal calprotectin ≥100 mg/kg (missing *n* = 85)	61 (34)	87 (38)
CRP ≥5 mg/L (missing *n* = 10)	50 (28)	76 (33)
Intra-abdominal surgery (CD)		98 (43)
Colectomy (UC)	10 (6)	
BPI–pain intensity, median (range)	7 (0–29)	7 (0–25)
BPI–pain interference, median (range)	10 (0–59)	8 (0–63)
HADS-D ≥8, *n* (%)	25 (14)	32 (14)
HADS-A ≥8, *n* (%)	61 (36)	72 (31)
25(OH) D, nmol/L, *n* (%)		
<50	78 (44)	125 (55)
50–75	76 (43)	69 (30)
>75	24 (13)	35 (15)
Current use of medication, *n* (%)		
Biologics	52 (29)	113 (50)
5-ASA	137 (77)	23 (10)
Prednisolone	26 (14)	19 (8)
AZA/MTX	46 (26)	87 (38)
Vitamin D supplementation	60 (34)	75 (33)

Abbreviations: UC: ulcerative colitis; CD: Crohn’s disease; SCCAI: Simple Clinical Colitis Activity Index; HBI: Harvey Bradshaw index; ASA: Aminosalicylic acid; AZA: Azathioprine; MTX: Methotrexate; CRP: C-reactive protein; HADS-D: Hospital Anxiety and depression—Depression; HADS-A: Hospital Anxiety and depression score—Anxiety; BPI: Brief Pain Inventory.

**Table 2:** Disease activity, depressive and anxiety symptoms by vitamin D level (nmol/L).

	25(OH) D <50 Median (Range)	25(OH) D 50–75 Median (Range)	25(OH) D >75 Median (Range)
UC (*n* = 178)			
SCCAI	5 (0–24)	4 (0–13)	4 (0–11)
CRP, mg/L	2 (0–215)	2 (0–53)	1 (0–35)
Calprotectin, mg/kg	102 (12–1672)	45 (24–1614)	71 (24–2052)
Depressive Symptoms (HADS-D)	3 (0–15)	3 (0–12)	2 (0–13)
Anxiety symptoms (HADS-A)	5 (0–17)	7 (0–17)	5 (0–15)
CD (*n* = 229)			
HBI	5 (0–24)	4 (0–13)	4 (0–11)
CRP, mg/L	3 (0–111)	2 (0–61)	2 (0–41)
Calprotectin, mg/kg	102 (24–1576)	67 (24–1299)	147 (24–2122)
Depressive Symptoms (HADS-D)	3 (0–13)	3 (0–14)	3 (0–10)
Anxiety symptoms (HADS-A)	5 (0–17)	7 (7–17)	5 (0–15)

Abbreviations: UC: ulcerative colitis; CD: Crohn’s disease; SCCAI: Simple Clinical Colitis Activity Index; HBI: Harvey Bradshaw index; CRP: C-reactive protein; HADS-D: Hospital Anxiety and depression score—Depression; HADS-A: Hospital Anxiety and depression score—Anxiety.

**Table 3:** Pain Severity and Localization of Pain in UC and CD Patients.

	UC (*n* = 178), *n* (%)	CD (*n* = 229), *n* (%)
Pain severity		
No pain	45 (25)	53 (23)
Mild pain	90 (51)	129 (56)
Moderate or severe pain	43 (24)	47 (21)
Pain localization		
Abdomen	60 (34)	96 (42)
Back	44 (25)	48 (21)
Joints	29 (16)	25 (11)
Pelvis	21 (12)	27 (12)

Pain severity: no pain, BPI score = 0; mild pain, BPI score 1–3; moderate or severe pain, BPI score ≥ 4.

**Table 4:** Univariate and Multivariate Analyses of Pain severity in UC (*n* = 178).

	Univariate OR (95% CI)		*p*-Value	Multivariate OR (95% CI)		*p*-Value
Age, years	1.02	0.99, 1.05	0.24			
Male (ref.)	1.0					
Female gender	3.34	1.86, 6.04	<0.01	2.84	1.57, 5.16	<0.01
25(OH) D <50 nmol/L (ref.)	1.0					
25(OH) D 50–75 nmol/L	1.33	0.56, 3.16	0.52			
25(OH) D >75 nmol/L	1.04	0.44, 2.48	0.93			
SCCAI ≥5	3.19	1.68, 6.04	<0.01	2.16	1.11, 4.22	0.02
CRP ≥5 mg/L	1.19	0.64, 2.20	0.59			
Calprotectin >100 mg/kg	1.31	0.70, 2.44	0.40			
Intra-abdominal surgery ^a^	1.22	0.51, 2.95	0.65			
Daily smoking	0.73	0.28, 1.93	0.53			
Depressive symptoms (HADS-D) ^b^	3.45	1.51, 7.85	<0.01	1.81	0.73, 4.53	0.21
Anxiety symptoms (HADS-A) ^b^	2.80	1.52, 5.16	<0.01	1.87	0.96, 3.67	0.07

Abbreviations: UC: ulcerative colitis; SCCAI: Simple Clinical Colitis Activity Index; CRP: C-reactive protein; OR: Odds ratio; CI: Confidence interval; HADS-D: Hospital Anxiety and depression score—Depression; HADS-A: Hospital Anxiety and depression score—Anxiety; ^a^ Colectomy —limited number of cases; ^b^ subscore ≥ 8.

**Table 5:** Univariate and Multivariate Analyses of Pain severity in CD (*n* = 229).

	Univariate OR (95% CI)		*p*-Value	Multivariate OR (95% CI)		*p*-Value
Age, years	0.99	0.97, 1.02	0.61			
Male (ref.)	1.0					
Female gender	2.23	1.33, 3.74	<0.01	2.09	1.23, 3.53	<0.01
25(OH) D <50 nmol/L (ref)	1.0					
25(OH) D 50–75 nmol/L	0.58	0.28, 1.19	0.14			
25(OH) D >75 nmol/L	0.66	0.30, 1.45	0.30			
HBI ≥ 5	2.36	1.40, 3.97	<0.01	1.97	1.14, 3.39	0.02
CRP ≥5 mg/L	1.48	0.87, 2.56	0.15			
Calprotectin >100 mg/kg	1.04	0.58, 1.84	0.90			
Intra-abdominal surgery ^a^	0.46	0.27, 0.77	<0.01	0.46	0.27, 0.77	<0.01
Daily Smoking	1.75	0.87, 3.56	0.12			
Depressive symptoms (HADS-D) ^b^	1.34	0.65, 2.77	0.43			
Anxiety symptoms (HADS-A) ^b^	1.66	0.96, 2.86	0.07	1.22	0.69, 2.16	0.50

Abbreviations: CD: Crohn’s disease., HBI: Harvey Bradshaw index., CRP: C-reactive protein., OR: Odds Ratio., CI: Confidence interval., HADS-D: Hospital Anxiety and depression score—Depression., HADS-A: Hospital Anxiety and depression score—Anxiety., ^a^ Bowel resection, stricturoplasty and lysis of adhesions; ^b^ subscore ≥ 8.

## Appendix A

### Further details on the method for analysis of 25-hydroxyvitamin D

25-hydroxyvitamin D [25(OH) D2 and 25(OH) D3] concentrations in serum were determined using a liquid chromatography–tandem mass spectrometry (LC-MS/MS) method, developed at the Hormone laboratory (Oslo University Hospital, Department of medical biochemistry, Norway). Deuterated [25(OH) D2 and 25(OH) D3] were used as an internal standard, of which 50 µL working solution 100 ng/mL 25(OH) D2-d3 and 25(OH) D3-d3) was added to 50 µL patient serum, calibrators or internal quality control samples. The samples were allowed to equilibrate for at least 10 min before the addition of 300 µL precipitation solution (2% NH_3_ in acetonitrile). The samples were left at −20 °C for 25 min to obtain satisfactory precipitation. The supernatant was transferred to a HybridSPE 96-well plate (Supelco, Bellefonte, PA, USA) for extraction. The solvent was removed by N_2_ (25 min, 70 °C) and the samples were reconstituted in 100 µL 50/50 MeOH/H_2_O (*v/v*). LC-MS/MS analysis was performed using a 1290 UHPLC system (Agilent, Santa Clara, CA, USA) coupled to a 6490 tandem mass spectrometer (Agilent). Of the sample, 20 µL were injected onto a 2.1 × 50 mm high strength silica-pentafluorophenyl (HSS-PFP) column (Waters, Milford MA, USA) heated to 40 °C. Mobile phase A consisted of 0.2% FA in water, while mobile phase B consisted of 0.2% FA in MeOH. A linear gradient from 72% B to 75% B in 1.5 min at a flow rate of 0.4 mL/min was used to separate and elute the analytes. Segmented acquisition directed interferences eluting during the first 0.8 min and last 1 min of the gradient into waste. Detection was performed with positive electrospray ionization and multiple reaction monitoring. Collision energies of 20V and 10V were used for fragmentation of 25(OH) D2/25(OH) D2-d3 and 25(OH) D3/25(OH) D3-d3, respectively. The ion transitions monitored for quantification of 25(OH) D2, 25(OH) D2-d3, 25(OH) D3 and 25(OH) D3-d3 were 395.3→269.1, 398.2→272.2, 383.3→257.2 and 386.8→257.0. Additionally, one or two ion transitions (qualifier ions) were monitored for the analytes and internal standards, respectively. The methods’ specificity was thus based on additional qualifier ions (and corresponding ion ratios) and the relative retention time of the analyte compared to the internal standard. The method limit of quantification was 17 nmol/L and 12 nmol/L, respectively, for 25(OH) D2 and 25(OH) D3. Excellent linearity was obtained in a range of 12.5–1248 nmol/L and 12.1–1211 nmol/L, respectively.
